# HPV and the Risk of HIV Acquisition in Women

**DOI:** 10.3389/fcimb.2022.814948

**Published:** 2022-02-10

**Authors:** Romaniya Zayats, Thomas T. Murooka, Lyle R. McKinnon

**Affiliations:** ^1^ Department of Immunology, Rady Faculty of Health Sciences, University of Manitoba, Winnipeg, MB, Canada; ^2^ Department of Medical Microbiology and Infectious Diseases, Rady Faculty of Health Sciences, University of Manitoba, Winnipeg, MB, Canada; ^3^ Centre for the AIDS Programme of Research in South Africa (CAPRISA), Durban, South Africa

**Keywords:** HPV, HIV, mucosal immunology, sexually transmitted infection, transmission

## Abstract

The risk of HIV acquisition is low on a per-contact basis but increased by transmission co-factors such as other sexually transmitted infections (STIs). Human papillomavirus (HPV) is a prevalent STI that most individuals will acquire HPV in their lifetime. Current HPV vaccines can prevent newly acquired infections, but are largely ineffective against established HPV, complicating worldwide eradication efforts. In addition to being the causative agent of cervical cancer, accumulating evidence suggests that HPV infection and/or accompanying cervical inflammation increase the risk of HIV infection in men and women. The fact that immunological features observed during HPV infection overlap with cellular and molecular pathways known to enhance HIV susceptibility underscore the potential interplay between these two viral infections that fuel their mutual spread. Here we review current insights into how HPV infection and the generation of anti-HPV immunity contribute to higher HIV transmission rates, and the impact of HPV on mucosal inflammation, immune cell trafficking, and epithelial barrier function.

## Introduction

HPV is the most common viral infection of reproductive tracts and the vast majority of sexually active individuals will acquire this virus at some point in their lives (Chesson et al., 2014). At 4 years following the first sexual intercourse, more than 50% of young women will become infected with a cervical HPV virus (Brianti et al., 2017). A majority of HPV infections are cleared without treatment within a few months of acquisition, and 90% of the infections are cleared within 2 years. Approximately 60% of infections will induce seroconversion and cervical samples will display mild (if any) abnormalities (Schiffman et al., 2007). However, in some cases of apparent HPV clearance it is believed that a state of latency is established, where immune control lowers viral replication to undetectable levels without achieving complete eradication (Liu et al., 2014; Shew et al., 2015; Fu et al., 2016). The difficulty in defining HPV infections as newly acquired, re-activation of latent virus, temporary deposition by an HPV-positive partner, or auto-inoculation from other sites, remains an epidemiological and clinical challenge (Strickler et al., 2005; Baay et al., 2011; Fu et al., 2015). While cervical lesions can be treated through electrocoagulation, cryotherapy, laser ablation or local surgery, when undiagnosed infection persists, there is an increased chance of developing pre-cancerous lesions (McCredie et al., 2008; Asiaf et al., 2014).

Despite availability of several effective, prophylactic HPV vaccines, the burden of HPV-associated pathology and cancer remains high (Kombe Kombe et al., 2020). The two most widely used prophylactic HPV vaccines are “Gardasil”, which provides protection against HPV 6, 11, 16, 18, 31, 33, 45, 52, and 58, and “Cervarix”, which provides protection against HPV 16 and 18 (Asiaf et al., 2014). These vaccines are prepared from purified virus-like particles (VLPs) of the highly immunogenic major capsid (L1) protein and delivered intramuscularly, inducing strong antibody responses in nearly all immunized individuals (Yang et al., 2004; Yan et al., 2005). While anti-HPV antibody responses during natural infection are poor, HPV vaccines are able to elicit high neutralizing antibody titres against the L1 capsid that are 10-10,000 times higher than during natural infection (Harro et al., 2001; Garland et al., 2007; Paavonen et al., 2009). Barriers to vaccine uptake, including misinformation, vaccine mistrust, social stigma, and vaccine availability, have limited many countries from reaching the recommended coverage of >80% (Bigaard and Franceschi, 2021). Additionally, the ability of HPV to induce latency complicates eradication efforts worldwide, as current vaccines are not effective against type-matched infections acquired prior to vaccination. Despite induction of strong cell-mediated immunity during HPV infection, it remains controversial whether sterilizing immunity is achieved or whether virus enters a latent state with the possibility of reactivating disease at a later time (Gravitt, 2012; Maglennon et al., 2014; Wang et al., 2015; Hinten et al., 2017). In this mini-review, we will discuss how HPV pathology and the underlying immune responses against infection alter the mucosal landscape that can favor acquisition of other STIs such as HIV. The ability of HPV to establish a chronic infection within the genital mucosal epithelium has implications on HIV acquisition risk and prevention strategies, and remains an area of active research, in particular with novel therapeutic vaccines reaching trial stages in 2021 (Ayesha et al., 2021).

## The Natural History of HPV Infection

Human papillomaviruses belong to the *Papillomaviridae* family and there are over 200 genotypes of HPV which are divided into low-risk HPV, causing anogenital and cutaneous warts or no obvious disease, and high-risk HPV, which in some cases lead to oropharyngeal and anogenital cancers (Forman et al., 2012; de Martel et al., 2012; Asiaf et al., 2014; Buchanan et al., 2016; de Martel et al., 2017; de Martel et al., 2020). Phylogenetically, HPV is classified into five genera (Bernard et al., 2010; Schiffman et al., 2016), with alpha HPV infecting mucocutaneous surfaces. While some alpha HPV present without pathology, others cause either highly productive warts or anogenital (cervical, vulvar, vaginal, penile, and anal) cancers (Brianti et al., 2017). HPV16 and HPV18 account for approximately 70% of all cervical cancers (Braaten and Laufer, 2008). HPV DNA has also been detected in oropharyngeal cancers, and some HPV have been discovered to contribute to cancers at cutaneous sites (Zhou et al., 2019). Many aspects of the natural history of HPV infection in women remain unclear due to the limitations of interpreting HPV infection status and practical limitations to conduct longitudinal studies spanning longer than 5-10 years. Typically, HPV is detected by identifying viral DNA in patient cervical samples, where DNA-negative women, tested by routine PCR, have been classified as “HPV uninfected”. Once HPV becomes undetectable, however, it is uncertain whether HPV is truly cleared, enters a latent state, or simply persists at low, undetectable levels. As the cervical epithelium constantly turns over, HPV latency may rapidly re-establish HPV infection. There is epidemiologic evidence that 3.3-19.4% of women who were observed to have cleared a specific HPV genotype subsequently experienced recurrent infection of the same HPV genotype (Strickler et al., 2005; Insinga et al., 2010; Trottier et al., 2010; Winer et al., 2011; Gravitt, 2012; Rodriguez et al., 2012). Periodical shedding of HPV (Liu et al., 2014), an increase in HPV prevalence during initiation of immune suppression (Hinten et al., 2017), and re-appearance of HPV following a negative test have all been observed (Polman et al., 2017). Additionally, whole cervix analysis utilizing the SPF10-PCR-DEIA-LiPA25 system, a broad-spectrum PCR assay which allows for amplification of 65-bp fragment in the L1 region from a wide range of HPV types, detected HPV in two patients who underwent hysterectomy unrelated to cervical abnormality and had tested HPV-negative. In patient 1, HPV31 was detected, whereas in patient 2, HPV18 and HPV53 were detected. The infection was very focal in both patients, and there was no progression to productive infection (Hammer et al., 2019). These data indicate that both patients had latent HPV infection without prior knowledge, underscoring the complexity of HPV infection in women.

## The HPV Life Cycle

**Figure 1:**
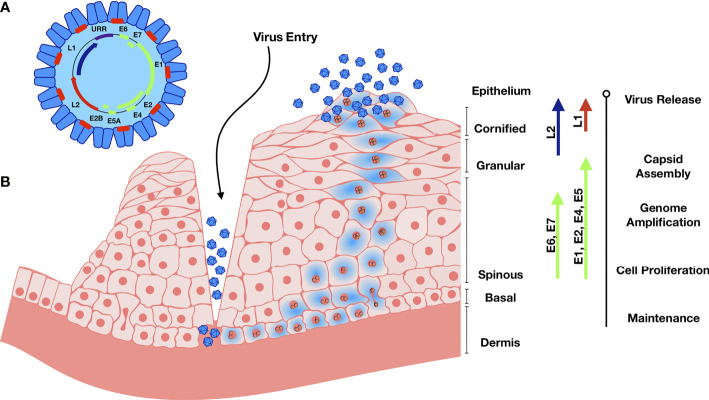
Genome organization and Life cycle of HPV. **(A)** The circular dsDNA genome is represented in the black circle. Position of early genome is represented by green, and L1 and L2 in blue and red respectively. URR – Upstream Regulatory Region. **(B)** Abrasion provides HPV access to basal keratinocytes. Viral DNA replication relies on the host DNA replication machinery and is supported by the early viral proteins E1 and E2. The viral genome replicates in the infected basal and stem cells and establishes HPV episome copies, which split between the daughter progeny as cells divide. The early viral proteins E6 and E7 stimulate the continued proliferation. Upon terminal cell differentiation in the upper epithelium, L1 and L2 become activated to package the high number of viral DNA copies. E4 disintegrates the cytokeratin filaments and the virus is released as keratinocyte remnants are sloughed off at the epithelial surface.

Human papilloma viral particles are 50-60 nm in diameter and lack a lipid envelope (**Figure 1**). Double stranded viral DNA is approximately 8 kbp, circular, packed with host histones and encapsulated by viral-encoded late proteins (Conway and Meyers, 2009). The HPV genome consists of 8 open reading frames, which can be divided into late (L) proteins, early (E) proteins, and an upstream regulatory region (URR) (Conway and Meyers, 2009). L1 and L2 encode for the major and minor capsid proteins, respectively. L1 consists of variable and constant regions, with the constant regions carrying the surface-specific antigenic epitopes that interact with the host membrane upon entry and are conserved among different HPV genotypes (Lowe et al., 2008; Dasgupta et al., 2011). E1 and E2 regulate the viral life cycle and viral genome replication, E4 regulates early viral gene products, and E6-7 cause cell cycle dysregulation and serve as oncogenes (Pinidis et al., 2016).

HPV has a unique mode of infection compared to other viruses. HPV reaches its target tissue, the basal cells of stratified squamous epithelium, only though micro-abrasions or epithelial trauma. Once the virions reach these cells, they cannot bind the cell surface receptor involved in internalization directly. After the basement membrane (BM) is exposed through epithelial trauma, HPV L1 major capsid protein binds the heparin sulfate proteoglycans (HSPGs) on BM (Schiller et al., 2010). This binding induces a cyclophilin B-dependent conformational change in the L1 3D structure, exposing the N-terminus of L2 minor capsid protein for cleavage by furin, a cellular proteinase (Richards et al., 2006; Bienkowska-Haba et al., 2009; Schiller et al., 2010). Proteolysis of L2 is followed by two subsequent interactions of L1 with HSPGs, resulting in further 3D conformational rearrangements of L1 and L2. These structural modifications result in the loss of bonds involved in the initial L1 interaction, resulting in viral movement to the keratinocyte zone and exposure of viral residues to the endocytic compartment to promote viral internalization by endocytosis (Schiller et al., 2010). While the infection occurs in non-differentiated cells, new viral progeny are produced primarily in terminally differentiated keratinocytes, while dividing basal cells serve as viral reservoirs (Meyers et al., 1992; McLaughlin-Drubin and Meyers, 2005; Conway and Meyers, 2009). Upon viral entry, the viral genome is transported into the nucleus and maintained as episomal DNA (McBride and Oliveira, 2006). Infection proceeds slowly, where viral transcription is initiated at 12-24 hours post-entry. Interestingly, when infected basal cells divide, two equally infected daughter cells are produced but contribute to disease progression differently: one remains in the basal layer while the other cell begins to differentiate and migrate through the upper layers of the epithelium (Lehman and Botchan, 1998; Bastien and McBride, 2000). During cell division, protein E2 tethers viral genomes to the cellular mitotic chromosomes, ensuring that the genome becomes enclosed within the nuclear envelope and segregated equally between the daughter cells (McBride and Oliveira, 2006). As the basal cell enters the differentiation process, proteins E1 and E2 form a complex with the viral DNA to recruit host DNA polymerase. E6 can bind the tumor suppressor p53 which promotes its ubiquitination and degradation (Rolfe et al., 1995). Protein E7 binds several host proteins, such as the retinoblastoma tumor suppressor family and cell-cycle regulatory proteins (Conway and Meyers, 2009). This modulation affects the cell cycle checkpoints, delays differentiation, and allows for HPV replication. E7 functions to maintain a cell mitotically active phenotype that drives expression of replication factors required for viral amplification that can reach thousands of copies per cell (Morris et al., 1993; Barksdale and Baker, 1993). Proteins L1 and L2 are then synthesized and are responsible for the viral DNA packaging and are only expressed in the terminally differentiated keratinocytes (Barksdale and Baker, 1993).

## Subversion of Host Immunity by HPV: Lessons Learned From Cervical Cancer

The HPV lifecycle is exclusively intraepithelial and new viral progeny are shed from the surface of the squamous epithelium. During the late phase of infection, infected cells increase expression of capsid proteins, which are highly immunogenic (Cason et al., 1989), but are shed quickly from the outer layers of the epithelium where there are low numbers of antigen presenting cells. Viral assembly and virion release from differentiated cells occur with minimal cytopathic effects, resulting in poor recognition by the host mucosal immunity. Protective anti-HPV antibodies only develop at 6-12 months after HPV infection, if at all, suggesting that T cells are responsible for controlling early viral replication (Carter et al., 1996). CD4^+^ T cells are of particular importance, exemplified by patients with regressed HPV^+^ lesions containing significantly higher numbers of HPV16 E6 and E7-specific CD4^+^ T cells (Kadish et al., 1997; Dillon et al., 2007; Kim et al., 2012). HIV^+^ individuals that have decreased CD4^+^ T cell numbers are susceptible to multiple HPV infections, prolonged viral persistence, and increased risk of cervical neoplasia (Ahdieh et al., 2001; Levi et al., 2004; Palefsky et al., 2006). Patients with high-grade cervical intraepithelial neoplasia have reduced Th1-type responses, and are often impaired (Welters et al., 2006), also supporting the notion that Th1 responses are crucial during HPV infection. Th1 responses are characterized by IFNγ and IL-2 production, and the lack of these cytokines is associated with persistent HPV infection and development of high-grade disease (Clerici et al., 1997; Dupuy et al., 1997; Scott et al., 1999). Interestingly, patients with high grade HPV^+^ lesions have a skewed Th2 response, a shift away from Th1 that may represent a mechanism employed by HPV to blunt host responses (Clerici et al., 1997; al-Saleh et al., 1998). Similarly, Th17 cells are primarily tumorigenic in the context of HPV infection and increased Th17 cell infiltration is associated with progression to invasive cervical cancer (Walch-Ruckheim et al., 2015; Xue et al., 2018). CD8^+^ T cells expressing α4β7 integrin, important for homing to mucosal sites, are present in healing lesions but reduced or absent in lesions which progress to cancer (McKenzie et al., 1991).

Viral nucleic acid sensing by TLR9 is thought to be important in the initial response to intracellular HPV by keratinocytes. Interestingly, TLR9 expression is highly upregulated by HPV-infected cells during low-risk HPV infection, but marginally during high-risk HPV (Cannella et al., 2015), suggesting blockade of this innate sensing pathway by virus. Additionally, TLR gene polymorphisms may contribute to cancer susceptibility: TLR2 del allele is significantly associated with cervical cancer susceptibility and TLR2 ins/del genotype is strongly associated with tobacco usage in women with cervical cancer, while TLR4 Thr/Ile genotype was significantly associated with the early stage of cervical cancer in North Indian women (Pandey et al., 2009). No association between TLR3 and TLR9 gene polymorphisms and risk of developing cervical cancer or tobacco usage was observed (Pandey et al., 2011).

**Figure 2:**
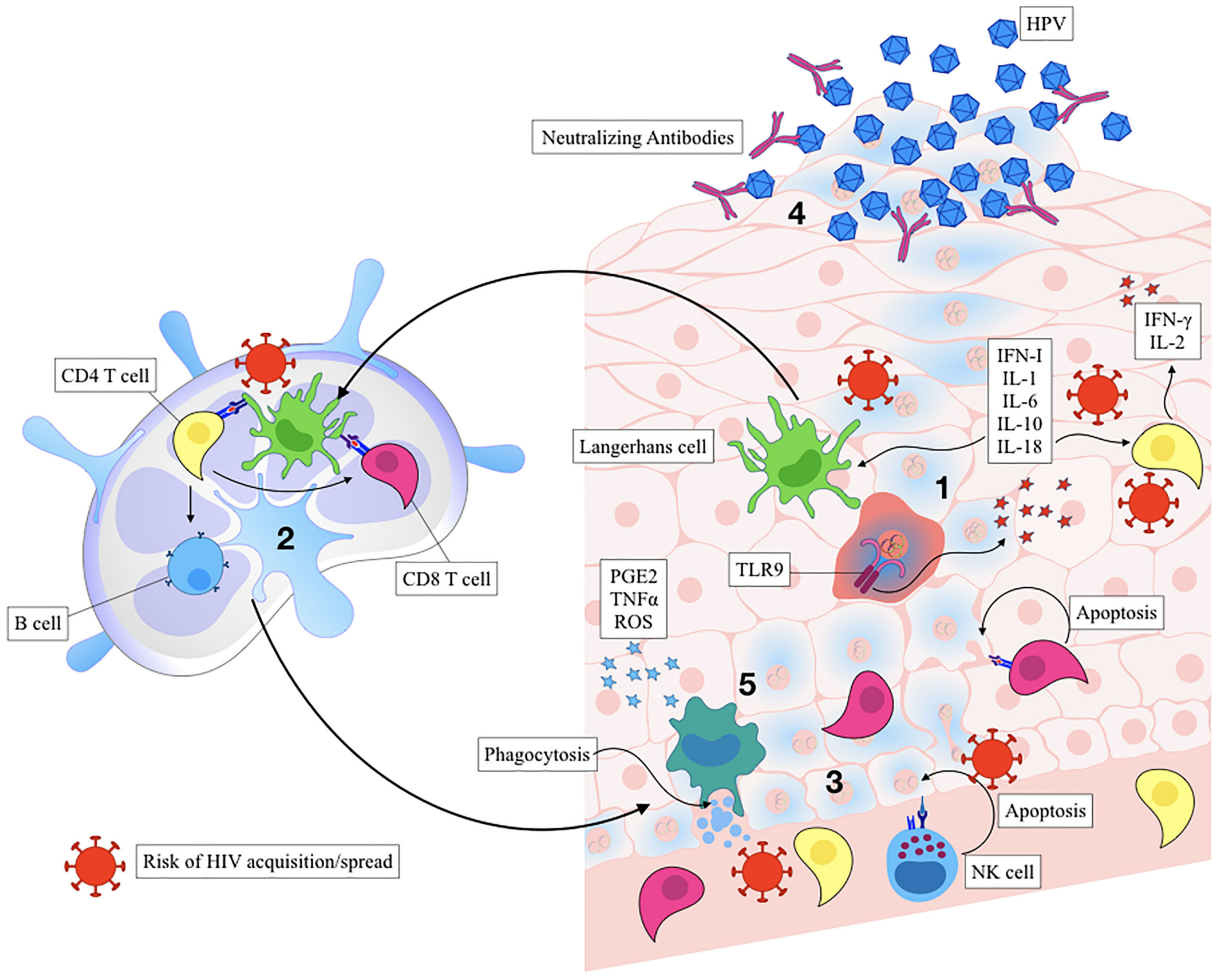
Immune control of HPV infection and the risk of HIV acquisition. Activated keratinocytes trigger the release of pro-inflammatory cytokines and chemokines leading to recruitment of Langerhans cells and other antigen-presenting cells (APCs) (Step 1). Inflammation is a known risk for HIV acquisition. Activated APCs process the antigen and migrate to the draining lymph node, where they help activate antigen specific CD8^+^, CD4^+^, and B cells (Step 2). HIV can utilize immune cell trafficking to spread from APCs to T Cells. The inflammatory state also activates innate immune responses, such as natural killer cells (Step 3), also associated with the increased risk of HIV acquisition. Inflammation drives infiltration of CD8^+^ T cells that kill viral-infected cells, and CD4^+^ T that contribute to viral clearance while also serving as HIV target cells. Plasma cells secrete neutralizing antibodies that function to neutralize cell-free HPV, preventing further infectious spread (Step 4). Macrophages and dendritic cells induce apoptosis and phagocytose apoptotic bodies, producing anti-inflammatory mediators and initiating the healing process (Step 5).

Activated keratinocytes produce antiviral and inflammatory mediators including type I interferons, IL-1, IL-6, IL-8, IL-10, IL-18, and TNF that function to recruit and stimulate a predominantly Th1-mediated immune response. Regional production of pro-inflammatory cytokines leads to the subsequent activation of Langerhans cells (LCs) and tissue resident macrophages and function to recruit CD4^+^, CD8^+^, and CD56^+^ cells to the site of lesion (Monnier-Benoit et al., 2006; Woo et al., 2008; Karim et al., 2013). As part of their immune evasion strategy, HPV-infected keratinocytes express low amounts of viral proteins to limit antigen presentation during the early phases of infection (Stoler et al., 1992). HPV oncoprotein E7 recruits various histone modification enzymes to suppress the transcription of TLR9 over time (Doorbar, 2005; Miller, 2008). E6 and E7 also block the pathogen recognition receptors (PRR) signal transduction, interfere with IFN-α/β receptor signalling pathway, and downregulate the NF-κB signaling pathway (Zhou et al., 2019). Lastly, HPV E7 can interact with and repress the MHC I promoter, while HPV E5 can reduce MHC I and CD1d expression by blocking the transport of these proteins to the cell surface (Zhou et al., 2019), limiting recognition by cytotoxic CD8 T cells. Together, these are several strategies employed by HPV to dampen host immunity and promote viral persistence and cancer progression. Interestingly, there are reports demonstrating that HPV can regulate the inflammatory response by manipulating the NF-κB signaling pathway and regulating the inflammatory cytokine cascade in order to induce chronic inflammation (Mangino et al., 2016). TLR9 levels have been recently demonstrated to be elevated in chronic HPV infections (Cannella et al., 2015), suggesting that TLR9 inhibition by virus may not be complete in some cases. In this scenario, sustained TLR9 expression without proper antiviral response can also drive chronic inflammation, leaving the epithelial barrier vulnerable to other infections and promote cancer progression (**Figure 2**) (Cannella et al., 2015).

Macrophages activated by regional pro-inflammatory cytokines can kill HPV-infected cells *via* tumor necrosis factor (TNF) (Routes et al., 2005) or antibody-dependent cytotoxicity mediated mechanisms. The oncogene E6 prevents the release of monocyte chemotactic protein-1 (MCP-1) by normal keratinocytes in response to TNF, impairing local macrophage recruitment during HPV infection (Hacke et al., 2010). High grade HPV^+^ lesions are characterized by high infiltration of predominantly M2-polarized tumor-associated macrophages (TAMs) (Hammes et al., 2007; Lepique et al., 2009) and are associated with poor prognosis (Lepique et al., 2009; Davis et al., 2016). M2 TAMs may differentiate from recruited monocytes within the immunosuppressive tumor microenvironment consisting of TGF-β, IL-10, IL-6, and PGE2 (Mantovani and Sica, 2010). It is speculated that M2 polarization of TAMs leads to an insufficient production of IL-12 that is necessary for NK, NKT, and Th1 cell function (Gabrilovich et al., 2012). Additionally, TAMs play an important role in recruitment of Tregs *via* CCL22, which are further maintained and stimulated *via* high levels of IL-10 (Gabrilovich et al., 2012).

Interference of innate immunity delays or impairs the induction of a robust adaptive immune response to HPV infection. Upon viral insult, Langerhans cells (LCs) that reside in the epidermis normally migrate to secondary lymphoid organs and prime the adaptive immune response. HPV has been shown to impair trafficking of APCs to the lymph node, delaying the onset of the immune response. Low infiltration of LCs into HPV-associated cancerous tumors is associated with disease severity (Caberg et al., 2009; Jiang and Xue, 2015). This is possibly due to downregulation of CCL20 through the inhibition of NF-κB signaling pathway (Guess and McCance, 2005; Le Borgne et al., 2006). Additionally, E-cadherin, which allows the LCs to remain in the epidermis to capture viral particles, is downregulated in HPV-associated tumors (Guess and McCance, 2005; Hubert et al., 2005) likely through E7-mediated promoter methylation. CCR7 expression on DCs was also observed to be downregulated, resulting in their reduced homing ability to lymph nodes (Laurson et al., 2010; Pahne-Zeppenfeld et al., 2014). Altogether, impairment of the LC trafficking to the lymph node can contribute to poor priming of effector T cells through reduced presentation capacity of viral antigens. Consequently, while CD8^+^ T cells infiltrate HPV-associated tumors, they do not seem to impair tumor growth, indicative of sub-optimal priming (Cromme et al., 1995).

Maturation of APCs is also impacted in HPV-infected tissues, as expression of MHC and co-stimulatory molecules CD80 and CD86 are downregulated (Bashaw et al., 2017). Many types of cancers establish an immunosuppressive environment that include expression of IL-10, TGF, IL-6, prostaglandin E2 (PGE2), and granulocyte-macrophage colony-stimulation factor (GM-CSF) that can reduce DC maturation potential (Herfs et al., 2009; Bayne et al., 2012; Torres-Poveda et al., 2014; Brencicova et al., 2017; Remes Lenicov et al., 2018). When HPV 16 E7-transgenic mouse grafts are grafted onto control mice, they do not get rejected due to recruitment of DCs expressing high levels of indoleamine 2,3-dioxygenase 1 (IDO1) (Mittal et al., 2013). The mechanism of IDO1-driven immunosuppression includes generation of regulatory T cells and suppressing effector responses by depleting tryptophan, which is important for T cell function (Kobayashi et al., 2008; Mittal et al., 2013). These studies are consistent with clinical observations that HPV^+^ cervical lesions express high levels of IDO1 (Kobayashi et al., 2008), suggesting that IDO1^+^ DCs may drive cancer progression by generating Tregs and/or inducing T cell anergy.

## Underlying HPV Infection Increase HIV Acquisition Risk

The per-exposure risk of HIV acquisition ranges from 0.08% for receptive vaginal sex (Boily et al., 2009) to 1.4% for receptive anal sex (Baggaley et al., 2010), but HIV transmission rates can be increased due to co-factors such as high viral load (VL), which increases the risk 26-fold (Wawer et al., 2005; Hollingsworth et al., 2008; Baeten et al., 2011). It is now well-recognized that high vaginal inflammation, defined by elevated concentrations of cervicovaginal cytokines, is strongly associated with elevated HIV acquisition risk, the underlying causes ranging from bacteria/viral sexually transmitted infections and a non-Lactobacillus, bacterial infections causing bacterial vaginosis (BV) (Ward and Ronn, 2010; Masson et al., 2015; Zevin et al., 2016; Liebenberg et al., 2017; Liebenberg et al., 2019). For example, cohort analyses of HIV-1-seronegative female sex workers in Nairobi, Kenya showed that genital ulcers and gonococcal infections are associated with an increased risk of HIV-seroconversion (Fowke et al., 1996; McKinnon et al., 2015). Similarly, HSV-2 infection is associated with a 3-fold increase in HIV acquisition risk in both men and women (Freeman et al., 2006). While high STI and HIV acquisition may be linked to increased exposures, underlying genital infections can independently increase HIV acquisition risk by disrupting mucosal barrier integrity, increased expression of neutrophil proteases and inducing an influx of CD4^+^ T cell populations in the genital epithelium and sub-mucosa, the prime targets of HIV during mucosal exposure (Arnold et al., 2016; McKinnon et al., 2018). Meta-analyses showed that a high proportion of HIV infections can be attributed to HPV infection of any genotype, associated with a near doubling of HIV acquisition risk in both men and women (Houlihan et al., 2012; Lissouba et al., 2013). A 2.4-fold increase in HIV infection was observed in individuals infected with either oncogenic or non-oncogenic HPV in sub-Saharan Africa, particularly in those with non-persistent infections (Averbach et al., 2010; Low et al., 2011). While biological explanations for how underlying HPV infections might increase HIV acquisition risk are incomplete, high CD4^+^ T cell numbers found within the stroma and epithelium are a marker for HPV lesion regression (Monnier-Benoit et al., 2006) and thereby increasing the number of susceptible target T cells for HIV (**Table 1**) (Mehandru et al., 2004; Veazey et al., 2008; McKinnon et al., 2011). Overlap in inflammatory mediators required for HPV clearance are also associated with high HIV acquisition risk, such as MCP-1, IL-8 and IP-10 (Masson et al., 2015; Arnold et al., 2016; Gosmann et al., 2017; McKinnon et al., 2018; Liebenberg et al., 2019). Increase in pro-inflammatory cytokine and neutrophil protease levels are associated with disruption in epithelial cell differentiation, cell-cell contacts, and epithelial barrier function and integrity (Kaul et al., 2008; Anahtar et al., 2015; Arnold et al., 2016). Increased Langerhans cells have also been shown to associate with HPV clearance (Shannon et al., 2017), and individuals with reduced LC numbers of develop HPV-positive cervical intraepithelial lesions (Mota et al., 1999). However, increased LC numbers may play a role in enhancing HIV susceptibility by binding HIV gp120 and facilitating transmission to CD4^+^ T cells (Arrighi et al., 2004; Koh et al., 2020). Finally, NK cells correlate with HPV clearance (Prata et al., 2015; Bjorkstrom et al., 2021), but NK cells were also shown to be biomarkers of increased HIV risk (Naranbhai et al., 2012).

**Table 1:** Low risk vs High risk HPV infection and HIV acquisition risk.

	Low risk HPV infection	High risk HPV infection
**Effects on basal epithelial level**	Basal cell proliferation is regulated by the presence of growth factors. Little to no E6/E7 expression.	E6/E7 expression stimulates cell cycle entry and cell proliferation in both the basal and parabasal epithelial layers, potentially leading to neoplasia.
**Primary role of E6/E7**	Facilitation of HPV genome amplification by stimulating cell cycle entry of the upper epithelial layers.	Facilitation of HPV genome amplification by stimulating cell cycle entry of the upper epithelial layers. Immune evasion.
**HIV acquisition risk in women with a prevalent HPV infection**	1.99	2.01
**HPV immune response and potential routes of entry for HIV**	Inflammation Natural killer cells influx Influx of Langerhans cells Influx of CD4^+^ T cells Disturbing of the mucosal barrier integrity	
**Public health opportunities of targeting HPV Immunology**	Immunotheraputic targeting of Toll-like Receptors-Interferons-Cycloxygenase-2-Tissue Inhibitor of Matrix Mettaloproteinases-Autophagy biochemical signal transduction cascades	

## Concluding Remarks

While HPV infection can cause cervical cancer in women, it is also a strong risk factor for HIV acquisition. This may have a biological basis; HPV infection induces genital inflammation, vaginal barrier disruption, and influx of activated target T cells, which have all been implicated in HIV risk. However, a clear mechanistic understanding of the factors that directly facilitate HIV acquisition is lacking. The fact that HPV can establish a latent state and re-establish infection complicates HIV prevention strategies in high-risk individuals, prompting the need for a better understanding of the cellular and molecular basis of anti-HPV immune control. Recent characterization of a mouse papillomavirus to study mucosal disease progression, host immune control and cervical cancer development *in vivo* would be an invaluable tool to address some of these outstanding questions (Cladel et al., 2017). Murine PV infection models can be integrated with other technologies, such as spatial transcriptomics analysis, to reveal how tissue heterogeneity and viral gene expression impacts host mucosal antiviral responses. These and other complementary approaches may identify common biological pathways activated by both HPV and HIV that can be therapeutically targeted to reduce HIV acquisition risk.

## Author Contributions

Conceptualization, writing and editing, RZ, TM, and LM. Figures, RZ. Supervision, TM and LM. Funding acquisition, TM and LM. All authors contributed to the article and approved the submitted version.

## Funding

This work was supported by the CIHR grant MRT-CDAA-159236 to TM and a studentship from Research Manitoba and University of Manitoba Student Fellowship to RZ. LM is supported by CIHR New Investigator Award.

## Conflict of Interest

The authors declare that the research was conducted in the absence of any commercial or financial relationships that could be construed as a potential conflict of interest.

## Publisher’s Note

All claims expressed in this article are solely those of the authors and do not necessarily represent those of their affiliated organizations, or those of the publisher, the editors and the reviewers. Any product that may be evaluated in this article, or claim that may be made by its manufacturer, is not guaranteed or endorsed by the publisher.
